# Psychometric Properties of the Anxiety About Aging Scale (AAS) in Lithuanian Adults

**DOI:** 10.1177/00914150241313359

**Published:** 2025-01-17

**Authors:** Goda Gegieckaitė, Karolina Petraškaitė, Olga Zamalijeva

**Affiliations:** 54694 Institute of Psychology at Vilnius University, Vilnius, Lithuania

**Keywords:** the Anxiety about Aging Scale, aging anxiety, Lithuanian, psychometric properties, measures

## Abstract

The Anxiety about Aging Scale (AAS) was developed by Lasher and Faulkender emphasizing a multidimensional approach to aging anxiety and addressing conceptual and psychometric issues of similar tools. Today this scale is one of the most used measures that allow to assess aging anxiety among younger and older populations. This study aimed to test the psychometric properties of the Lithuanian version of the AAS. A total sample of 528 Lithuanian adults, ages ranging from 18 to 82 (*M* (*SD*) = 33.6 (14.5)); 22.7% male, were included in this study. Results showed that the Lithuanian version of AAS had good psychometric characteristics. Using confirmatory factor analysis, the four-factor structure originally proposed by Lasher and Faulkender was confirmed. The total AAS scale and all four subscales showed good internal consistency. This study confirmed that the Lithuanian version of AAS can be used in further studies as suggested by scale authors.

## Introduction

Not only is every living person growing older, but Western societies are aging as well, with an increasing part of the population becoming older adults. Answers to questions about how to age successfully are as relevant as ever. Successful aging is a complex topic as age-related changes are related not only to genetic factors or health-related behaviors, but various psychological factors might also have a cumulative effect in later life. Thoughts about aging and being older can elicit anxiety in some people and studies have shown that attitudes and emotions about aging seem to be related to people's well-being (Bryant et al., 2012; Seow et al., 2022) and that prior negative attitudes towards aging were associated with more negative future aging experience (Tully-Wilson et al., 2021; Westerhof et al., 2023). It is important to understand what helps people to have a healthier relationship with their aging.

To study these questions, we need to have valid aging anxiety measures. Lasher and Faulkender (1993) addressed this issue by developing the Anxiety about Aging Scale (AAS). AAS is one of the most often used and validated scales for aging anxiety in younger populations (Faudzi et al., 2019), but its application is not necessarily limited to measuring aging anxiety among younger adults because it allows evaluation of attitudes to present and future aging even in later life (Sargent-Cox et al., 2014). Lasher and Faulkender (1993) developed this scale aiming to consider the issues identified in previous scales, mainly the construct of aging anxiety being vaguely defined and inconsistent and scales lacking the evidence of validity and reliability data. Authors emphasized a multidimensional approach to aging anxiety and using the literature of gerontology distinguished four important areas of anxiety about aging: physical, psychological, social, and spiritual. They theorized that each of these dimensions involves three separate types of fears—fear of aging, fear of being old, and fear of old people. Authors generated 84 items based on these categories, out of which 20 items formed 4 factors—Fear of Old People, Psychological Concerns, Physical Appearance, and Fear of Loss. Fear of Old People factor measures reaction to older people. Lasher and Faulkender hypothesized, that this factor measures a more defensive, covert aging anxiety that manifests by negative reactions to older people and may reveal aging anxiety that is not necessarily evident in other factors. The factor of Psychological Concerns was described by the scale's authors as addressing important psychological tasks from Erikson's psychosocial development theory one needs to solve for successful aging, for example, satisfaction about already lived life and finding contentment, staying active and autonomous in the current stage of life (Erikson et al., 1986; Perry et al., 2015). The Physical Appearance was explained as measuring anxiety about physical looks changing with age. The Fear of Loss factor measured anxiety about something being taken away or lost in old age and while related to the idea represented in the Psychological Concerns factor, Lasher and Faulkender (1993) described the items of Fear of Loss as being more externally focused.

According to Lasher and Faulkender (1993), the factor structure they obtained from their data is consistent with the literature and has good face validity. A more detailed analysis of the original scale validation data on 312 adults across different age groups showed sufficient psychometric properties, Cronbach alpha for the whole scale was .82 and varying from .69 to .78 for the subscales, factor loadings to the intended factors ranging from .47 to .79 and correlations between factors ranged from .20 to .39 (Lasher & Faulkender, 1993). The authors also found that aging anxiety was higher among men, was negatively associated with age, and had a significant correlation with the amount and quality of contact with older people, misinformation about aging, and self-efficacy. Scale's authors emphasized that further studies replicating the four-factor structure are needed to establish the construct validity of the AAS more firmly.

Although thirty years have passed since the development of the AAS, the interest in validating AAS in other countries and languages has only begun to increase in the last decade. AAS have been validated in Australia (Sargent-Cox et al., 2014), translated and adapted in Taiwan (Gao, 2012), Greece (Koukouli et al., 2014), Spain (Fernández-Jiménez et al., 2020), Mexico (Zueck-Enríquez et al., 2021), among Iranian adults (Pakpour et al., 2021), Turkey (Kalaycı, 2021), Malaysia (Din & Minhat, 2021), Croatia (Hošnjak & Goriup, 2024) and Pakistan (Afsar et al., 2024). In all these studies, confirmatory factor analyses or other alternatives to test factor structure were performed, and the four-factor structure originally suggested by Lasher and Faulkender (1993) was confirmed. Additionally, invariance was tested between gender (Fernández-Jiménez et al., 2020; Gao, 2012; Pakpour et al., 2021; Sargent-Cox et al., 2014) and age groups (Gao, 2012; Sargent-Cox et al., 2014). While some invariance can be noticed, for example, Sargent-Cox et al. (2014) found some invariance of the Fear of Loss factor between older and younger participants, but according to authors they were not major and should not be over-interpreted, therefore overall, the AAS showed satisfactory invariance between different age and gender groups in mentioned studies.

However, in these validation studies, some items were found to be problematic and were removed from some of the translated and adapted versions. Item 4 (“I have never lied about my age in order to appear younger”) from the Physical Appearance subscale is often found to have low factor loading or other problems and sometimes is removed from the scale because of it (Din & Minhat, 2021; Gao, 2012; Kalaycı, 2021; Zueck-Enríquez et al., 2021). This might suggest that lying about age might not be a good indicator of aging anxiety or might be not a common response. Items 5 (“I fear it will be very hard for me to find contentment in old age”) from the Psychological Concerns subscale and 17 (“I am afraid that there will be no meaning in life when I am old”) from the Fear of Loss subscale sometimes produce less than desirable results as well and need to be cross-correlated with other items, factors or to be removed altogether (Din & Minhat, 2021; Gao, 2012; Koukouli et al., 2014; Sargent-Cox et al., 2014; Zueck-Enríquez et al., 2021). Items 5 and 7 seem to be related in meaning even though they represent different factors, so it might explain why they need to be cross-loaded on each other's factors or load lower on their intended factors. More items, for example, item 20 (Physical Appearance), item 13 (Fear of Old People), or others, have also shown inconsistent outcomes (Gao, 2012; Koukouli et al., 2014).

Cronbach's alphas of the full scale and subscales seem to be similar across studies ranging from around .67 to .88 (Fernández-Jiménez et al., 2020; Gao., 2012; Pakpouri et al., 2021; Sargent-Cox et al., 2014), even though some items are removed in some versions of the scale. However, more variation between different validation studies can be seen in the correlations between factors. In some studies, it may be noticeably low and ranging from .03 to .32 (Din & Minhat, 2021), while in some others it ranges from .32 to .55 (Fernández-Jiménez et al., 2020) or even .51 to .66 (Afsar et al., 2024). However, the interpretation of these differences is harder and might be partially explained by some versions of the scale removing items that are intercorrelated between factors, while others do not. However, some of the found differences in intercorrelations of the factors or even differences in items’ performances might be affected by some cultural differences. For example, the Fear of Old People might have a lower or higher association with other factors, depending on cultural traditions of intergenerational living arrangements or the ageism level in the country. People's perceptions of older age and anxiety about aging might also be affected by the living conditions of the older generation in that country (De Paula Couto et al., 2022).

Having a valid measure to study aging anxiety in the Lithuanian population is a relevant issue as problems such as social exclusion and poverty in the older adult population are significantly higher than in the general population (Eurostat, 2019) and ageism is high compared to Western European countries, more similar to that of Eastern and Central Europe and Mediterranean countries (Rapolienė, 2015). According to the World Happiness Report (Helliwell et al., 2024), there is a substantial gap between younger and older cohorts in Lithuania, as people over 60 are ranking 44 positions lower in happiness than the ones under 30, indicating a likely difference not only in perception but also the actual life circumstances of the older population in the country. Similarly to other European countries ageing is not regarded favorably in Lithuania. It is viewed as related to a loss of social status, and despite the public declaration of respect and admiration of the high morals of older adults, in reality, older people are ignored and discriminated against (Rapolienė, 2015). Moreover, compared to other European countries Lithuanian older adults are some of the most likely to see age as a factor limiting their autonomy (OECD, 2022) and the biggest proportion of the general population would be really uncomfortable with having a high-ranking elected official older than 75 (Rychtaříková, 2019). On the other hand, lack of more detailed and newer international research does not allow accurate conclusions about specific views about aging in Lithuania. While previously described data indicate the possibility of higher ageism, it seems rather unlikely there being unique Lithuanian aging stereotypes or ageism that would manifest in significantly different form than in other European or Western countries.

Aging anxiety and negative perceptions of aging seem to be related to worse aging outcomes (Tully-Wilson et al., 2021; Westerhof et al., 2023), and a better understanding of factors contributing to or mitigating aging anxiety might be an important stepping stone in helping people with their aging anxiety and better aging projection. What is more, negative reactions toward older adults, resulting in subsequent social isolation, and ageism in various areas including the labor market, may in part be explained by aging anxiety manifesting as fear of older people (Lasher & Faulkender, 1993), further explorations of determinants and other factors related to aging anxiety in aging societies may provide additional insight into this issue and offer new perspectives in battling ageism. The well-being of the older population is an extremely important issue as the Lithuanian population is projected to be aging fast and to have the sixth highest old-age dependency ratio in Europe by 2050 (Eurostat, 2024). Therefore, it is important to study attitudes toward aging in Lithuania, and to do that we need a valid and reliable tool measuring aging anxiety. The aim of this study is to test the psychometric properties of the Lithuanian AAS.

## Methods

### Procedure and Participants

The final sample of the study was 528 Lithuanian adults. Participants’ age ranged from 18 to 82 (*M *= 33.63, *SD *= 14.47), 401 (75.9%) were women, 120 (22.7%) were men, and 7 (1.3%) reported other gender identification. Detailed demographic information of the participants is presented in Table 1.

**Table 1.** Participant Characteristics (*N* = 528).

Gender, *n* (%)	
Men	120 (22.7%)
Women	401 (75.9%)
Other	7 (1.3%)
Age (years)	
Mean (*SD*)	33.63 (14.47)
Range	18–82
Age groups, *n* (%)	
18–29	257 (48.9%)
30–39	111 (21.1%)
40–49	77 (14.6%)
50–59	45 (8.6%)
60 and more	36 (6.8%)
Education level, *n* (%)	
Higher university education	254 (48.2%)
Higher non university education	49 (9.3%)
Unfinished higher education or currently studying	152 (28.8%)
Secondary education	67 (12.7%)
Basic or lower education	5 (0.9%)
Residency type, *n* (%)	
Big city	339 (64.2%)
City	113 (21.4%)
Town	34 (6.4%)
Village	42 (8%)
Marital status, *n* (%)	
Married or cohabitating with a partner	279 (52.8%)
Partnering without cohabitation	55 (10.4%)
Single	162 (30.7%)
Divorced	18 (3.4%)
Widow/widower	14 (2.7%)
Has children, *n* (%)	
Yes	215 (40.7%)
No	313 (59.3%)
Occupation, *n* (%)	
Employed	344 (65.2%)
Unemployed	67 (12.7%)
Retired	30 (5.7%)
Student	213 (40.3%)
Other	20 (3.8%)
Has chronic illnesses, *n* (%)	
Yes	116 (22.0%)
No	412 (78.0%)

*Note.* Valid percentages excluding missing cases are shown.

The study was reviewed and approved by the Institutional Committee on Research Ethics in Psychology. Data collection took place from January to the beginning of April 2024 using a website specifically developed for this study. The participants were given information about the study through an informed consent form and after agreeing to participate, were directed to the online questionnaire. The recruitment was implemented through online advertisements shared on social media sites like Facebook, as well as sharing it to different public groups. The study was introduced as a survey about people's attitudes about aging and death as this study was part of a bigger project to validate both aging and death anxiety measures. The only inclusion criteria were participants being at least 18 years old and since informed consent and questionnaire were in Lithuanian language, they needed to have a good understanding of the Lithuanian language. In total, 540 participants finished the online questionnaire, out of which 12 were excluded from the study because of the failing of at least one of the two attention checks. Attention checks asked participants to mark specific numbers on the Likert scale.

### Measures

#### Anxiety About Aging Scale

AAS (Lasher & Faulkender, 1993) has 20 items, which are divided into 4 factors all containing 5 items: Fear of Old People (e.g., “I enjoy talking with old people”), Psychological Concerns (e.g.,“I expect to feel good about life when I am old”), Physical Appearance (e.g.,“I have never dreaded looking old”), and Fear of Loss (e.g.,“I worry that people will ignore me when I am old”). The participants are asked to rate each item on a 5-point Likert scale, indicating the level of agreement with each statement. The scores of positively worded items then were reversed in the Lithuanian version of the scale. The total scale and subscale scores are obtained by summing the corresponding items and a higher score indicates higher aging anxiety.

#### Translation and Adaptation of the AAS Into Lithuanian

Lithuanian version of the AAS was prepared according to Lasher and Faulkender's (1993) original article, describing the scale's development, and other scale validations studies (Fernández-Jiménez et al., 2020; Gao, 2012; Pakpour et al., 2021; Sargent-Cox et al., 2014; Zueck-Enríquez et al., 2021). Scale items were independently translated by two authors, Lithuanian native speakers, psychologists having a good command of English and Lithuanian languages, and experience in previous adaptations and validations of English language scales to the Lithuanian language. These translations were then compared and after additional discussions a semi-final version of the scale was prepared. This version of the scale was given to 5 people, who were requested to explain their understanding of the items and to comment on the clarity of each of them. After this phase, the formulation of some items was adjusted for better clarity, and the final version of the scale was prepared.

While Lasher and Faulkander (1993) do not explicitly specify the direction of response anchors of a 5-point Likert scale, from the authors reported reversed items and their interpretation of the results, it can be understood that in their Likert scale, lower scores might have indicated higher agreement with statements, a higher total score indicating higher aging anxiety. However, according to the majority of other international AAS versions (Fernández-Jiménez et al., 2020; Gao, 2012; Pakpour et al., 2021; Zueck-Enríquez et al., 2021), and in order to provide a more intuitive scale design, in a Lithuanian version of AAS Likert scale ranged from 1 (strongly disagree) to 5 (strongly agree) and then positively phrased item scores were reversed, so that the higher AAS score would indicate higher levels aging anxiety. The response anchors of ‘strongly disagree’ and ‘strongly agree’ were adapted to the more culturally common Lithuanian equivalents, which can be translated back to English as ‘completely disagree’ and ‘completely agree’. Lasher and Faulkander (1993) describe items 5, 2, 6, 8, 14, and 17 as negatively worded, however, it seems in their original article they accidentally left out item 20, which is also negatively worded. In the Lithuanian version, other positively worded items’ scores were reversed and added with negatively worded items’ scores for a total score of the AAS and its four subscales.

### Aging Anxiety Frequency Items

Additionally, to test how the AAS score correlates with similar items, for this study we developed four items measuring the frequency of the occurrence of the aging anxiety in participants’ lives. The items were “I think about my aging,” “I feel anxiety when thinking about aging,” “I check for aging signs,” and “I am afraid of my aging.” These items were given to participants on a separate scale after the AAS. The participants were asked to rate how often they were doing things described in the items (1, very rarely; 2, rarely; 3, sometimes; 4, often; 5, very often).

### Fear of Death Items

Aging anxiety is known to be related to death anxiety, so for this study, we developed two items about death anxiety. One item was “Rate, how much in your life you think about the fact, that you will die someday”, answer options ranging from 1 (“I almost never think about it”) to 7 (“I think about it constantly”). The second item was “Rate, how much are you afraid of your death”, with answer options ranging from 1 (“I am not afraid at all”) to 7 (“I am very afraid”).

### Big Five Inventory–Neuroticism

For the discriminant validity analysis, the Neuroticism subscale from the Big Five Inventory was included (John et al., 2008). The subscale consists of 8 items (e.g., “I see myself as someone who gets nervous easily”), that are rated on a 5-point Likert scale ranging from 1 (strongly disagree) to 5 (strongly agree). Scale previously had satisfactory psychometric properties (John et al., 2008) and in this study, Cronbach alpha shows good internal consistency -.81.

### Data Analysis

Psychometric properties were tested by calculating means, skewness, and kurtosis first for each item in the scale and after confirming the factor structure, for total AAS and each subscale.

**Figure 1.**
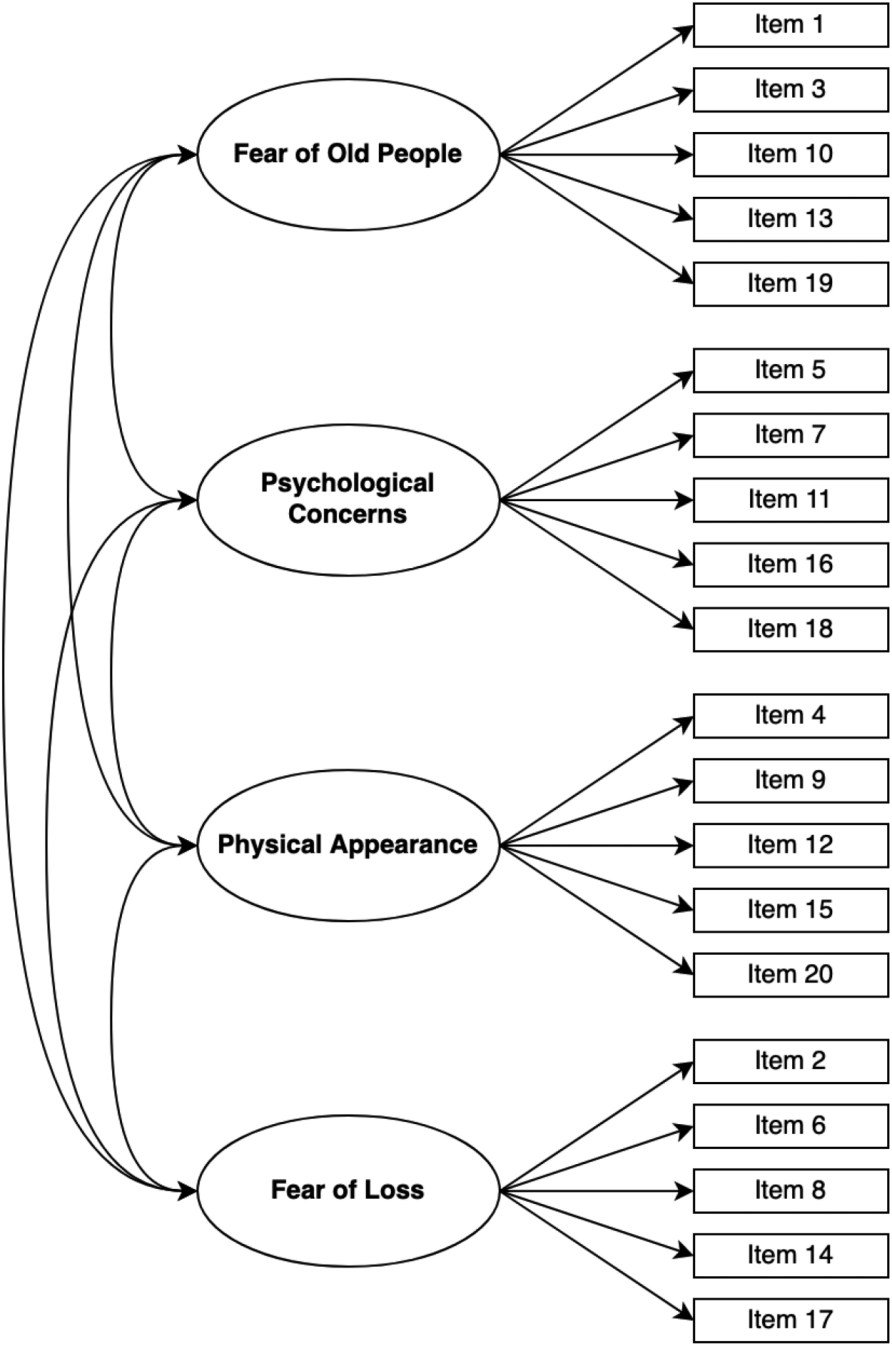
Anxiety about Aging Scale's factor structure proposed by Lasher and Faulkender (1993) and tested in this study.

Confirmatory factor analysis (CFA) testing proposed original AAS factor structure by Lasher and Faulkender (1993) (Figure 1) was performed. Lavaan package in R, using the Rstudio program, was used to perform calculations for CFA. Scaled indices from R were reported as main indices, robust indices were additionally included in reporting of the results as well.

CFA model estimation was performed using a maximum likelihood estimator with a robust standard errors (MLR) estimator. While some suggest that the WLSMV estimator should be used for Likert scales (DiStefano & Morgan, 2014) as they are technically ordinal data, however it was also shown that when the scale has 5 and more points, MLR estimation performs equally well and in specific cases, like non-normal distribution, may outperform estimators traditionally used for categorical indicators, and with small and medium samples (around 500 people) MLR and WLSMV both have strengths and weaknesses in different areas (Li, 2016). To assess the model's goodness of fit, indices of the Chi-square (*χ*^2^) test, Comparative Fit Index (CFI), Tucker–Lewis Index (TLI), and Root Mean Square Error of Approximation (RMSEA) were used. Indexes were considered satisfactory when CFI and TLI were above .9, and RMSEA below .08 (Hu & Bentler, 1999; Marsh et al., 2004). Chi-square being non-significant was preferable, however, as this index is sensitive to sample size and model complexity it was not considered deciding for the goodness of model fit determination. Factor loadings of items were considered to be sufficient when they were above .40 (Brown, 2015).

The properties of internal consistency in this study were assessed by providing Cronbach's alpha coefficients for total AAS and each subscale, as well as the Pearson correlations between each of the subscales and the AAS score. The analysis was also done to calculate Cronbach's alpha of each subscale and total AAS scale if each of the item was deleted, as well as correlations of each item to the total AAS and each subscale. As not all items were normally distributed, Spearman correlations were used to calculate relations between items and total AAS and subscales. According to Cohen's (1988) recommendations correlations above .10 were considered small, correlations above .30 were considered medium, and correlations above .50 were considered large or strong.

Finally, additional items that were developed to assess aging anxiety and fear of death can be considered ordinal variables, therefore Spearman correlations were calculated to evaluate their association with total AAS and subscales, while Pearson correlations were calculated between total AAS, subscales, and neuroticism. Descriptive information of the neuroticism scale (BFI-N) and items for aging anxiety frequency and death anxiety can be seen in Table A1 in the Appendix.

## Results

An analysis of each scale's item showed that means for most of the items ranged from 2.09 to 2.84, with the only exceptions of item 4 (*M *= 1.44, *SD *= 1.09) and item 6 (*M *= 3.20, *SD *= 1.26). Skewness for all items ranged from ±0.04 to ±0.64, except for item 4 (“I have never lied about my age in order to appear younger”) from the Physical Appearance subscale, whose skewness was 2.49. Kurtosis ranged for all items from ±0.15 to ±1.24, except for item 4, whose kurtosis value was 4.80.

Next confirmatory factor analysis analyzing the four-factor model proposed by Lasher and Faulkender (1993) was performed. Factor loadings and factor correlations of CFA are shown in Table 2. Item 4 had low, yet significant, factor loading on the Physical Appearance factor (.12) all other factors had sufficient factor loadings (.44–.85). Fear of Loss and Fear of Old People factors had the lowest correlation (.15), and Fear of Loss and Psychological Concerns factors had a high correlation (.80). The goodness of model fit indices were showing a satisfactory fit: (*χ*^2^ (164) = 418.035, *p *< .001; CFI = 0.925, TLI = 0.913, and RMSEA = 0.054 (90% CI = 0.048–0.060)). Additionally, robust indices reported in R were also satisfactory—CFI = 0.928, TLI = 0.916, and RMSEA = 0.057 (90% CI = 0.051–0.064).

**Table 2.** Items’ Factor Loadings and Factor Correlations From CFA.

	First order model
Fear of Old People	
Item 1	.85
Item 3	.67
Item 10	.87
Item 13	.70
Item 19	.71
Psychological Concerns	
Item 5	.71
Item 7	.53
Item11	.67
Item 16	.51
Item 18	.79
Physical Appearance	
Item 4	.12
Item 9	.78
Item 12	.78
Item 15	.82
Item 20	.44
Fear of Loss	
Item 2	.65
Item 6	.51
Item 8	.51
Item 14	.71
Item 17	.67
	Factors’ correlations
Fear of Old People and	
Psychological Concerns	.34
Physical Appearance	.32
Fear of Loss	.15
Psychological Concerns and	
Physical Appearance	.64
Fear of Loss	.80
Physical Appearance and Fear of Loss	.59

*Note*. All factors loading and correlations reported in the table were significant at *p* < .01 level, except item 4, that was significant at *p* = .010.

Analysis of descriptive statistics of the total scale and subscales are reported in Table 3 and correlations between subscales and total scale are in Table 4. Cronbach alpha for the total scale and subscales were good ranging from .72 to .87. Kurtosis ranged from ±0.71 to ±0.5, and skewness ranged from 0.04 to 0.33. The Fear of Old People subscale had the lowest correlation with the total AAS score (.58), while other subscales’ correlation with the total score ranged from .77 to .82. Fear of Old People also had the lowest correlations with other subscales (*r* = .13–.29) and Fear of Loss and Psychological Concerns had the highest correlation with each other (*r* = .62).

**Table 3.** Descriptive Statistics of the AAS and the Four Subscales.

	Mean (*SD*)	*α*	Min–max	Kurtosis	Skewness
Total AAS	50.91 (12.62)	.87	22–93	0.05	0.11
Fear of Old People	12.61 (4.28)	.87	5–25	−0.15	0.33
Psychological Concerns	12.20 (3.88)	.78	5–25	−0.27	0.19
Physical Appearance	12.09 (4.49)	.72	5–24	−0.71	0.24
Fear of Loss	14.00 (4.52)	.74	5–25	−0.60	0.04

**Table 4.** Correlations Between Subscales and the Total AAS.

	Total AAS	Fear of Old People	Psychological Concerns	Physical Appearance
Fear of Old People	.58**			
Psychological Concerns	.82**	.29**		
Physical Appearance	.79**	.28**	.53**	
Fear of Loss	.77**	.13**	.62**	.48**

*Note.* ** *p* < .01.

Analysis of Cronbach's alphas of total AAS and each subscale if each item was deleted mostly did not reveal any problematic items, except for item 4, which deletion would improve the Physical Appearance subscale's Cronbach's alpha from .72 to .79. Also, from all of the items, item 4 had the lowest correlation to the total AAS score (*r *= .19, *p *< .001), other items’ correlations ranged from .36 to .69 (*p *< .001). Each item had the highest correlations to the theoretically intended subscale (*r *= .32–.86, *p *< .001), although intended subscale scores do include the item itself, so these correlations might be inflated. However, item 5 had a correlation of .76 to its intended Psychological Concerns subscale, but also a .63 correlation to the Fear of Loss scale. Item 17 had a correlation of .66 to its intended Fear of Loss subscale but also a correlation of .62 to the Psychological Concerns scale.

Finally, correlations between the total AAS score, its subscales, and items measuring the frequency of different aging anxiety aspects, death anxiety items, and neuroticism were analyzed (Table 5). Items measuring the frequency of feeling anxiety when thinking about aging and fear of own aging had strong positive correlations to AAS and items indicating how often they think about aging and checking for aging signs had lower, medium strength positive correlations with the total AAS score. Higher death anxiety and thinking about death were associated with higher aging anxiety. Being afraid of death had a higher positive correlation to total AAS than the frequency of thinking about dying someday. The Fear of Old People subscale compared to other subscales of AAS had the lowest correlations to all items about aging anxiety frequency and death anxiety. Notably, the frequency of being afraid of own aging had the highest positive correlation with the Physical Appearance subscale. Neuroticism had a moderately strong positive correlation to the total AAS score and three AAS subscales, but its correlation to the Fear of Old People subscale was not significant.

**Table 5.** Correlations Between the Total AAS Score, its Subscales and Neuroticism, and Questions About Aging Anxiety, Death Anxiety.

	Total AAS	Fear of Old People	Psychological Concerns	Physical Appearance	Fear of Loss
Thinking about own aging	.36**	.10*	.24**	.34**	.34**
Anxiety thinking about aging	.62**	.17**	.48**	.59**	.56**
Checking for aging signs	.39**	.10*	.27**	.40**	.34**
Afraid of own aging	.66**	.18**	.50**	.65**	.55**
Thinking about dying someday	.27**	.04	.24**	.24**	.26**
Afraid of death	.46**	.11*	.36**	.40**	.46**
Neuroticism	.40**	.07	.46**	.29**	.37**

*Note*. * *p* < .05; ** *p* < .01.

## Discussion

This study aimed to assess the psychometric properties of the Lithuanian version of the AAS developed by Lasher and Faulkender (1993). The results of CFA showed the inner structure of the Lithuanian AAS in general corresponds to the four-factor model proposed by Lasher and Faulkender (1993). This study provides evidence that the scale measures four distinct dimensions of aging anxiety: Fear of Old People, Psychological Concerns, Physical Appearance, and Fear of Loss.

A more detailed analysis of the Lithuanian AAS properties revealed similar characteristics to the original Lasher and Faulkender (1993) version and other versions validated in other languages and cultures. When analyzing specific items of the scale, some were found to be somewhat more problematic. Just like in many previous validation studies, item 4 was shown to be not performing as well (Din & Minhat, 2021; Gao, 2012; Kalaycı, 2021; Zueck-Enríquez et al., 2021). This item was shown to have the lowest mean compared to other items, meaning, that most people did not agree that they have lied about their age to appear younger. This item also had the lowest factor loading in CFA and had the lowest correlation to the total AAS score. However, even with this item, the factor model tested in CFA showed sufficient model fit indices, and for the purposes of being consistent with the original and other not modified versions of the scale this item was left in the final model. The fact that item 4 seems to have lower loading in many validation studies in different countries—Malaysia, Taiwan, Turkey, and Mexico (Din & Minhat, 2021; Gao, 2012; Kalaycı, 2021; Zueck-Enríquez et al., 2021), but also in original Lasher and Faulkender's (1993) and adaptation study in Australia (Sargent-Cox et al., 2014), might be indicating that lying about one's age is a weaker indicator of aging anxiety among different cultures. On the other hand, item 4 did not seem to have lower factor loading in Spanish validation (Fernández-Jiménez et al., 2020) and among Iranian adults (Pakpour et al., 2021), so there might be some specific cultural aspects in some countries after all.

Items 5 and 17 in the analysis showed a similar strength correlation to one other subscale than the one they belong to, showing that they might be related to more factors. These items had been found to have problems in some of the previous studies (Din & Minhat, 2021; Gao, 2012; Koukouli et al., 2014; Sargent-Cox et al., 2014; Zueck-Enríquez et al., 2021). Item 17, originally intended for the Fear of Loss factor, indicates concern about not finding meaning in life when a person will be old, while item 5, asks about not finding contentment in older age and is meant for the Psychological Concern subscale. It seems theoretically reasonable that these two items seem to have also similarly high correlations to both factors, as these subscales describe similar, although subtly distinct aspects of aging anxiety. It shows participants do not necessarily understand the aspect of finding contentment and meaning in life in older age as one distinctly internal psychological experience and the other as more of an external loss. The fact that items 5 and 17 were correlating or cross-loading to other factors in some of the other countries as well (Kalayci, 2021; Gao, 2012; Sargent-Cox et al., 2014), once again shows that these aspects are understood similarly among at least a few different cultures. In CFA these items showed high factor loadings on their theoretical factors and were confirmed to fit well into the factor structure proposed by Lasher and Faulkender (1993). While a more detailed analysis of the items showed some weaker points, overall, a four-factor model with all items in the original factors as suggested by Lasher and Faulkender (1993), shows a good model fit and we suggest that the Lithuanian version of the scale can be used in the original form, with all originally proposed items for each subscale and total AAS score.

Internal consistency shown by Cronbach's alphas of the scale and subscales were satisfactory, ranging from .72 to .87 similar to other studies where it ranged from .67 to .88 (Fernández-Jiménez et al., 2020; Gao, 2012; Pakpouri et al., 2021; Sargent-Cox et al., 2014). As in the original Lasher and Faulkender (1993) study, the Fear of Old People subscale had the lowest correlation with the total AAS score, while others had higher and similar correlations with the total AAS. However, intercorrelations between factors revealed that in our study they were on the higher side compared to previous findings. While in Lasher and Faulkender's (1993) original study, the highest correlation was also between Fear of Loss and Psychological Concerns, it demonstrated only medium strength (.39), while in our study it was high (.62). In later validation studies, correlations between factors vary quite a lot, with some reporting even small to medium strength correlations (Din & Minhat, 2021). However, some of the studies also found correlations between the factors of AAS being high (Afsar et al., 2024; Fernández-Jiménez et al., 2020). Even with some of the correlations between the four factors in this study being high, the model tested in CFA showed that four distinct factors measuring aging anxiety fit the data well.

Total AAS score had a medium correlation with neuroticism and fear of death, both constructs theoretically and empirically found to be related to aging anxiety (Allan et al., 2014; Barnett and Adams, 2018; Bodner et al., 2015). Neuroticism was associated with the Psychological Concerns, the Physical Appearance, and the Fear of Loss, which can be explained by the tendency for people with higher neuroticism to experience more negative emotions, so feel more anxiety over the aging process and interpret information more negatively, therefore, to interpret older age as a more negative experience. The slightly stronger association of neuroticism with the dimension about concerns how a person will feel psychologically when they are older might be related to people with neuroticism not only overestimating the negativity of the future but also forecasting their realistic tendency to feel more negative emotions. It has been found that people with higher neuroticism accurately predict that they will feel more negative emotions in the future or after a specific event than their peers (Hoerger et al., 2016; Kong, 2015) and other studies also show that they also overestimate how much negative emotions they will feel in the future (Kong, 2015).

Fear of death had the strongest association with the Fear of Loss dimension. This might be interpreted as people similarly fearing losing something that they find valuable in life now either in old age or through their death. Fear of Old people had only a weak association with fear of death and no association with neuroticism. Lasher and Faulkender (1993) theorized that this dimension might show a more unconscious aging anxiety. However, these weak or non-significant associations with neuroticism and fear of death might also suggest, that this dimension might have different underlying mechanisms compared to other dimensions and have other important contributing factors. As it assesses prejudiced reactions and discrimination towards older people, this dimension theoretically overlaps with the ageism construct (Ayalon et al., 2019), therefore factors related to ageism might have stronger associations with this dimension.

We were also interested to see how AAS will be associated with the frequency of different behaviors, cognitions, and emotions related to aging anxiety. We found that the AAS had the highest positive associations with how often participants are afraid of their aging and feel anxiety when they think about aging, which is very similar to what the AAS is supposed to measure—the aging anxiety frequency and negative attitudes about aging. However, there were only medium positive associations between aging anxiety and how often people think about aging and check for aging signs. While there is still an association between the two, this seems to show that people might not be thinking a lot about aging and still experience aging anxiety or be thinking about aging anxiety more often, but not necessarily feel a lot of aging anxiety. Some people with high aging anxiety also might purposefully avoid thinking about aging as a distressing topic, as higher anxiety symptoms have been repeatedly linked to experiential avoidance (Akbari et al., 2022).

When considering possible cultural effects on our study results, similarities of the findings to the other studies and cultures are more evident than their differences. We found that items most often having problems in adaptations of the scale for other cultures were behaving very similarly in our study. This specificity indicates that aging anxiety aspects might be similar in its contents across cultures. For example, De Paula Couto et al. (2022) found that young and old people stereotypes were mostly similar across the USA, Germany, and India, indicating that cultural differences were not as big as could have been expected. Previous studies show that Lithuanians show higher discrimination of older people in some respects compared to some of the Western Europe countries (OECD, 2022; Rapolienė, 2015), but the AAS scores show participants rating their aging anxiety around the middle of the scale and mean of AAS score is somewhat similar to that found by Lasher and Faulkender (1993). However, for true aging anxiety level comparisons further studies designed for cross-cultural comparisons should be done with representative samples. Only higher correlations between factors might show Lithuanians’ aging anxiety aspects being more closely related to each other compared to other cultures. It is hard to explain the cultural reason for this result and methodological aspects should also be kept in mind, for example, several other studies removed intercorrelated items, which might decrease correlations between factors.

This study had several limitations that might have affected the results and need to be addressed when interpreting the findings. First of all, the sample of the study was not representative, almost half of the sample were people aged between 18 and 29, mostly women and with a higher education. While the previous studies shown that the AAS showed satisfactory invariance between different age and gender groups (Fernández-Jiménez et al., 2020; Gao, 2012; Pakpour et al., 2021; Sargent-Cox et al., 2014), some of the items in the previous studies were shown to be understood differently by different age groups and gender (Gao, 2012; Sargent-Cox et al., 2014) and it might affect some of the factor loadings and other results we found in this study. Because of the unequal proportions of participants in different gender and age groups categories in this study, we did not test the invariances between groups ourselves. Also, when testing the AAS associations with the death anxiety and the frequency of some specific aging anxiety aspects, we only used single-item measures, that were created for this study. Therefore, correlations may have been detected due to the wording of the items, but not necessarily be indicative of true relationships to the attitudes these items were aimed to represent. We did not include other validated aging anxiety measures to test true convergent validity, however, to our knowledge there were not any such measures adapted in the Lithuanian language. Measures in our study were not counterbalanced and the order of scales in the questionnaire could have created some response biases, however, the AAS was the first scale in the study after demographic questions to minimize possible effects of other scales. These limitations need to be kept in mind when interpreting the results of our study, however, they do not undermine important information obtained about the psychometric properties of the Lithuanian version of the AAS.

## Conclusion

This study shows that the Lithuanian version of the AAS had good psychometric properties, the factor structure fit the original model proposed by Lasher and Faulkender (1993) and can be used in further studies using total and subscales scores as originally suggested by the scale authors.

Previous studies have shown that the older population in Lithuania experiences significant social problems and society has a high level of ageism (Eurostat, 2019; Rapolienė, 2015), more research on the Lithuanian population's attitudes toward aging and aging anxiety is needed in order to move toward understanding factors contributing to better relationship with aging experience and possibilities to improve the relationship with one's aging. This matter is especially important as previous studies have shown that more profound negative attitudes toward aging are related to poor mental and physical outcomes in the aging process (Tully-Wilson et al., 2021; Westerhof et al., 2023). Having a validated tool to research aging anxiety in the Lithuanian population is one step forward toward this goal.

## Appendix A

**Table A1.** Descriptive Statistics of Items About Aging Anxiety Frequency, Death Anxiety and BFI-N (Neuroticism).

	Mean (*SD*)	Min–max	Kurtosis	Skewness
Thinking about own aging	2.64 (1.02)	1–5	−0.54	−0.04
Anxiety thinking about aging	2.37 (1.15)	1–5	−0.76	0.38
Checking for aging signs	2.10 (1.10)	1–5	−0.43	0.67
Afraid of own aging	2.44 (1.17)	1–5	−0.72	0.36
Thinking about dying someday	3.43 (1.59)	1–7	−0.75	0.16
Afraid of death	3.72 (1.97)	1−7	−1.13	0.29
BFI-N (Neuroticism)	3.26 (0.75)	1.25–5	−0.37	−0.12
